# *Aldanella attleborensis* (Mollusca) from Cambrian Stage 2 of the Three Gorges Area and Its Stratigraphic Implications

**DOI:** 10.3390/biology12020261

**Published:** 2023-02-07

**Authors:** Yaqin Qiang, Junfeng Guo, Guoxiang Li, Zuchen Song, Jiaxin Peng, Jie Sun, Jian Han, Zhifei Zhang

**Affiliations:** 1 Key Laboratory of Western Mineral Resources and Geological Engineering, Ministry of Education, School of Earth Science and Resources, Chang’an University, Xi’an 710054, China; 2 State Key Laboratory of Palaeobiology and Stratigraphy, Nanjing Institute of Geology and Palaeontology and Center for Excellence in Life and Paleoenvironment, Chinese Academy of Sciences, Nanjing 210008, China; 3 Shaanxi Key Laboratory of Early Life and Environments, Department of Geology, Northwest University, Xi’an 710069, China

**Keywords:** *Aldanella attleborensis*, taxonomic revision, ontogeny, stratigraphic correlation, Cambrian Stage 2, South China

## Abstract

**Simple Summary:**

*Aldanella attleborensis*, is a mollusk fossil found worldwide; its first appearance datum (FAD) has been suggested as one of the potential markers for defining the base of Cambrian Stage 2. However, *A. attleborensis* was uncommonly recovered in the Cambrian strata of South China, and if occurring, was usually described as *Aldanella yanjiaheensis* or an indeterminate species in the literature due to lack of detailed taxonomic work. That is an obvious obstacle to correlate and subdivide the Cambrian strata in South China and worldwide. Recently, numerous specimens of *Aldanella* were collected from Member 5 of the Yanjiahe Formation in the Three Gorges area, South China, which is the type locality of *A. yanjiaheensis*. Morphological and taxonomic study of these specimens supports that *A. yanjiaheensis* is a junior synonym of *A. attleborensis*. Shell microstructure and muscle scar imprints on its internal mold surface indicate that *A. attleborensis* may belong to the gastropods. The occurrence of *A. attleborensis* in Member 5 of the Yanjiahe Formation suggests that this stratigraphic unit shall be of Cambrian Stage 2 in age, and can be correlated with the Dahai Member of the Zhujiaqing Formation in Yunnan, uppermost Kuanchuanpu Formation in Shaanxi, and the uppermost Tianzhushan Member of the Dengying Formation in Hubei, South China. Meanwhile, using the FAD of *A. attleborensis* as a bio-marker, the upper Terreneuvian strata in South China, Siberia, Avalonia, and Estonia can be well correlated.

**Abstract:**

Some small shelly fossils are important index fossils for global stratigraphic subdivisions and correlations of the Cambrian Terreneuvian. The first appearance datum (FAD) of the cosmopolitan mollusk *Aldanella attleborensis* has been suggested as one of the potential markers for defining the base of Cambrian Stage 2. *Aldanella* fossils were uncommon in South China, and if occurring, were often described as *Aldanella yanjiaheensis*, *A. attleborensis*, or indeterminate species in the literature, while *A. yanjiaheensis* was often taken as a junior synonym of *A. attleborensis*. Nevertheless, a detailed taxonomic revision of *A. yanjiaheensis* based on material from its type locality awaits to be made. In this study, we systematically re-evaluated *A. yanjiaheensis* based on the numerous specimens collected from the base of Member 5 of the Yanjiahe Formation in the Three Gorges area, western Hubei Province of South China. Detailed taxonomic comparison further substantiates that *A. yanjiaheensis* is a junior synonym of *A. attleborensis*, signifying its strong potential for a global correlation across paleocontinents. Morphological parameter analyses indicate that the length and width of shell tube of *A. attleborensis* shows allometric growth. The nearly cosmopolitan distribution and characteristic morphology of *A. attleborensis* indicate that it can play a significant role in the subdivision and correlation of Cambrian Stage 2. The co-occurrence of *A. attleborensis* and *Watsonella crosbyi* from the base of Member 5 of the Yanjiahe Formation corroborates that Member 5 belongs to Cambrian Stage 2.

## 1. Introduction

The Cambrian radiation is one of the most significant bio-revolutions in Earth’s history, and the sudden appearance of Small Shelly Fossils (SSFs) represents a crucial episode of this radiation event [1,2,3]. Microscopic mollusks, which are the major components of SSF assemblages, are important for studying the origin and early evolution of mollusks [4,5,6,7,8] as well as for the subdivision and correlation of pre-trilobitic Cambrian strata [4,9]. At present, the definition of the base of Cambrian Stage 2 (Terreneuvian Series) has not been determined yet. Given the cosmopolitan distribution of two molluscan taxa in Cambrian Terreneuvian strata, the first appearance datum (FAD) of *Aldanella attleborensis* Shaler and Foerste, 1888 [10] or *Watsonella crosbyi* Grabau, 1900 [11], has been suggested as a potential marker for defining the base of Cambrian Stage 2 [12,13,14,15,16,17,18,19,20,21,22].

*Aldanella* Vostokova, 1962 [23], with its dextral or sinistral, turbospirally coiled shell, is a characteristic mollusk with morphological variation and has a worldwide distribution in the late Terreneuvian strata. Although its systematic position is controversial (gastropods, helcionelloids, hyoliths, or polychaetes), *Aldanella* has been more often considered as the earliest gastropod [13,24,25,26,27]. Owing to the over-evaluation of the morphological variations, 15 species had been described under this genus on the basis of worldwide materials [10,13,28,29,30,31,32,33,34,35,36,37,38]. After several rounds of taxonomic revisions during the last few decades [13,39,40,41], the genus *Aldanella* was suggested to include only six valid species: *A. attleborensis* (type species), *A. crassa*, *A. golubevi*, *A. utchurica*, *A. operosa*, and *A. sibirica* [13]. However, the supposedly diagnostic features of the different species (e.g., conch height, whorl expansion rate, and whorl number) were still considered to be within the range of intraspecific variation by Landing et al. [41].

Although there are numerous mollusk records in the Cambrian Terreneuvian strata on the Yangtze Platform (e.g., eastern Yunnan, western Hubei, southern Shaanxi, and southwestern Sichuan), the occurrence of *Aldanella* is quite scarce. Owing to the scarcity and poor preservation of specimens, the importance of *Aldanella* in stratigraphy has not been well studied in South China. The first report of *Aldanella* in South China was from the Yanjiahe Formation in the western Hubei Province, and a new species, *Aldanella yanjiaheensis* Chen, 1984 was erected [33]. Subsequently, *A. yanjiaheensis* (or described as an indeterminate species or *A*. *attleborensis*) was sporadically recovered in other localities in China, such as the Dahai Member of the Zhujiaqing Formation of Yunnan [9,42], Kuanchuanpu Formation of Shaanxi [43], Yanjiahe Formation [19,20,44,45,46] and Huangshandong Member (=Tianzhushan Member) of the Dengying Formation [47] of Hubei, and Yurtus Formation of Tarim [48,49]. Although *A. yanjiaheensis* was tentatively regarded as a junior synonym of *A. attleborensis* based on the morphological features depicted in the low-resolution illustrations in the literature [13,39,41], a detailed comparative study has not been conducted systematically yet because there was no re-examination of the type specimens and no new specimens were recovered from the type locality until recently. Consequently, the taxonomic status of *A. yanjiaheensis* is not formally resolved yet and the species requires a detailed taxonomic revision based on more specimens from the Yanjiahe Formation [50].

Recently, hundreds of specimens of *Aldanella* were collected from the Yanjiahe Formation in the Three Gorges Area. Based on these new materials, the purpose of this study is to give a detailed description of these specimens and to investigate the taxonomy of *Aldanella* in order to formally verify whether *A. yanjiaheensis* is a junior synonym of *A. attleborensis*. Additionally, we will highlight the stratigraphic importance of *A. attleborensis* in South China, which is also taken as a potential index fossil for defining the base of the Cambrian Stage 2 like *Watsonella crosbyi*.

## 2. Geological Setting, Materials, and Methods

All the specimens in this study were collected from the base of Member 5 of the Yanjiahe Formation in the Yanjiahe and Gunziao sections, Yichang, Hubei province (for the geological setting, see figure 1 in [19] and figure 2 in [20]). The Yanjiahe Formation is approximately 40 m thick and is mainly exposed around the southern and western flanks of the Huangling anticline [19,20,46,51]. Overlain by the Shuijingtuo Formation, the Yanjiahe Formation disconformably overlies the Baimatuo Member of the Dengying Formation (Ediacaran), and can be lithologically subdivided into five members (originally described as 5 beds, see [19]). The SSFs of the Yanjiahe Formation were mainly recovered in Members 2, 3, and 5. Member 2 is approximately 1.9 m thick, and consists of siliceous-phosphatic, intraclastic dolostone with SSFs belonging to the *Anabarites trisulcatus*–*Protohertzina anabarica* Assemblage Zone [19,33]. Member 3 (15.7 m thick) consists of gray-black limestone interbedded with silty shale, and yields SSFs of the *Purella antiqua* Assemblage Zone in siliceous-phosphatic nodules [19]. In addition, the silty shale of this member is abundant in macrofossils, such as *Protoconites minor* and *Yanjiahella biscarpa* [52,53,54]. Member 5 (1.1 m thick) consists of siliceous-phosphatic, intraclastic limestone, and yields SSFs of the *Watsonella crosbyi* Assemblage Zone (or *Aldanella attleborensis* Assemblage Zone) [20]. Although there was no chronology documentation of the Yanjiahe Formation, the U–Pb age from the base of the Shuijingtuo Formation in the Yangtze Gorges area gives an age of 526.4 ± 5.4 Ma [55], and the Yanjiahe Formation was suggested as recording the biogeochemical history of approximately the first 15 Ma of the Cambrian Period [56]. Overlain by the Shuijingtuo Formation, the age of the Yanjiahe Formation must be older than the upper Shuijingtuo Formation. According to the age of base of Cambrian Stage 2 in the International Chronostratigraphic Chart (ca. 529 Ma), herein estimates the age of Member 5 of the Yanjiahe Formation approximately 529 Ma based on the co-occurring of *A. attleborensis* and *W. crosbyi*. Abundant SSFs have been found in Member 5 of the Yanjiahe Formation, such as cnidarians (*Septuconularia yanjiaheensis* [51], *Octapyrgites elongatus* [57], *Decimoconularia isofacialis* [58], *Septuconularia crassiformis* [59]), mollusks (*Watsonella*, [20], *Anabarella* [20,60], *Oelandiella* [20], *Igorella* [19,60]), and sclerites [61], as well as a mass of tubes, embryos, and shells [unpublished data].

Approximately 10 tons of rock samples were dissolved in 10% acetic acid. The insoluble residues were washed and collected using sieves. More than 100 specimens of *Aldanella* were manually picked from the residues using a binocular microscope. Selected specimens were coated with gold and photographed using an FEI Quanta 650 Scanning Electron Microscope (SEM). Up to 1000 micro-computed tomographic (micro-CT) images (.tiff format) of the specimen CUBar22-4 were obtained using a Zeiss Xradia 520 at the State Key Laboratory of Continental Dynamics, Northwest University, Xi’an, China. The micro-CT data were processed using VG Studio 3.0 Max for 3D volume rendering.

**Figure 1:**
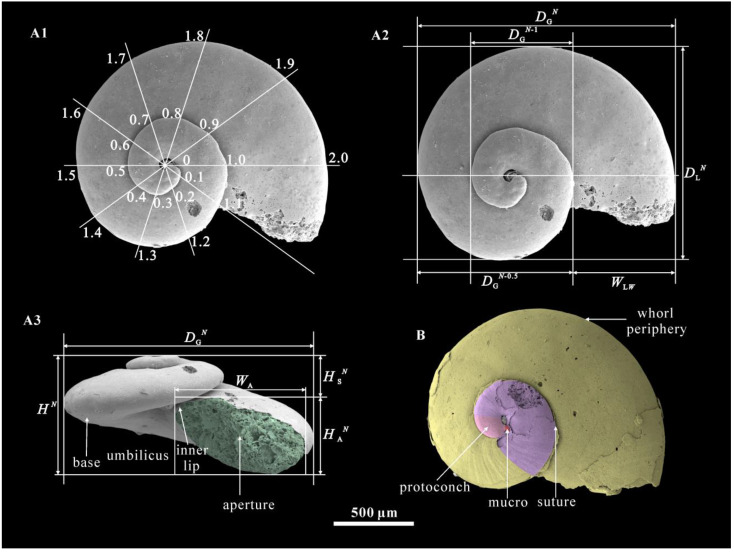
Scheme of measurements and anatomical terminology for *Aldanella attleborensis*. (**A**), internal molds, CUBar186-11; (**A1**), the measurement scheme of number of whorls, (**A2**), the measurement scheme of diameter, (**A3**), the measurement scheme of height; (**B**), internal mold, CUBar250-2. Different color definitions: green—aperture; red—mucro; pink—protoconch; yellow—last whorl; pink and purple—spire. Symbols and abbreviations are as follows: *D*_G_*^N^*—greater shell diameter at the stage of *N* whorls; *D*_L_*^N^*—lesser shell diameter at the stage of *N* whorls; *D*_G_*^N^*^−0.5^—greater shell diameter at the stage of *N*−0.5 whorls; *D*_G_*^N^*^−1^—greater diameter at the stage of *N*−1; H*^N^*—shell height at the stage of *N* whorls. *H*_S_*^N^*—spire height at the stage of *N* whorls; *H*_A_*^N^*—height of aperture at the stage of *N* whorls; *W*_A_—aperture width; *W*_LW_—width of shell tube of the last whorl; K^exp^—ratio of greater shell diameter to the diameter of the previous whorl; K—ratio of shell height to greater diameter of shell; K^iso^—ratio of greater to lesser diameters of shell.

All the specimens were cataloged and deposited at Chang’an University (CU), Xi’an, China. The scheme of measurements (Figure 1A1–A3) and anatomical terminology (Figure 1A3,B) used herein follow those of Parkhaev and Karlova [13].

## 3. Results

### Systematic Palaeontology

Phylum Mollusca Cuvier, 1797 [62]

Class Gastropoda Cuvier, 1797 [62]

Order Pelagielliformes Mackinnon, 1985 [63]

Family Aldanellidae Linsley and Kier, 1984 [64]

Genus *Aldanella* Vostokova, 1962 [23]

1962 *Aldanella* Vostokova, pp. 65–66 [23].

2011 *Aldanella* Vostokova; Parkhaev and Karlova, p. 1174 (and synonymy list therein) [13].

2017 *Aldanella* Vostokova; Kouchinsky et al., p. 345 [21].

Type species. *Pleurotomaria* (*Raphistoma*) *attleborensis* Shaler and Foerste, 1888 [10]; Cambrian Stage 2; Hoppin Hill, Massachusetts, USA.

Species included. Fossils of *Aldanella* have been reported widely from the lower Cambrian of the main continents. Owing to considerable morphological variation, 15 species had been erected under *Aldanella* (or its junior synonym), including *A. attleborensis* Shaler and Foerste, 1888 [10], *A. crassa* Missarzhevsky in Rozanov et al., 1969 [30], *A. golubevi* Parkhaev, 2007 [38], *A. utchurica* Missarzhevsky in Rozanov et al., 1969 [30], *A. operosa* Missarzhevsky in Rozanov and Missarzhevsky, 1966 [29], *A. rozanovi* Missarzhevsky in Rozanov and Missarzhevsky, 1966 [29], *A. kolymica* Barskova, 1988 [34], *A. costata* Missarzhevsky, 1989 [35], *A. patelliforma* Bokova, 1990 [36], *A. kunda* Öpik, 1926 [28], *A. polonica* Lendzion, 1977 [32], *A. yanjiaheensis* Chen, 1984 [33], *A. iberica* Gubanov in Gubanov et al., 2004 [37], *A. sibirica* Parkhaev and Karlova, 2011 [13], and *Paraaldanella kotuica* Golubev, 1976 [31]. Through a detailed examination of these species, Parkhaev and Karlova [13] considered existence of species over-splitting under *Aldanella* and made a detailed taxonomic revision of these species. After their revision, *Aldanella* included six valid species, i.e., *A. attleborensis*, *A. crassa*, *A. golubevi*, *A. utchurica*, *A. operosa,* and *A. sibirica*, while *A. yanjiaheensis* was considered a junior synonym of the type species.

Emended diagnosis. (After [13]) Shell small, low-conical, turbospirally coiled, dextral or sinistral. Whorl surface flattened or slightly convex, and periphery rounded. Aperture oval in outline, oblique to periphery. Spire projecting onto the last whorl surface. Umbilicus varying in width, with or without opening umbilical perforation. Small, cap-shaped protoconch faintly separated from teleoconch. One or two septa occasionally developed in spire. Shell ornamented with growth lines and folds.

Remarks. The genus *Aldanella* was originally established by Vostokova in 1962 [23] based on material from Siberian Platform and on previous figured specimens from Massachusetts while the latter was inappropriately filed under the genus *Pleurotomaria* [10]. Although the morphology was very similar to that of *Aldanella*, Golubev [31] erected the genus *Paraaldanella* (type species *P. kotuica*) based on specimens from the western Anabar region. Parkhaev and Karlova [13] regarded the diagnostic features of *P. kotuica* within the intraspecific variation of *Aldanella utchurica*, and considered *P. kotuica* as a junior subjective synonym of *A. utchurica*. Therefore, *Paraaldanella* has been treated as a junior synonym of *Aldanella*. The low-conical shell of *Aldanella* is similar to that of dextral *Philoxenella* Vostokova, 1962 [23] and sinistral *Barskovia* Golubev, 1976 [31], but the difference is that both *Philoxenella* and *Barskovia* have circular apertures. From the apical view, *Aldanella* is similar to *Pelagiella* Matthew, 1895 [65], but the difference is that the latter has a flattened spire, and a larger, approximately triangular aperture.

Occurrence. Cambrian Fortunian to Stage 3, South China, Tarim, Siberia, North America, Europe, Mongolia (?), Australia.

**Figure 2:**
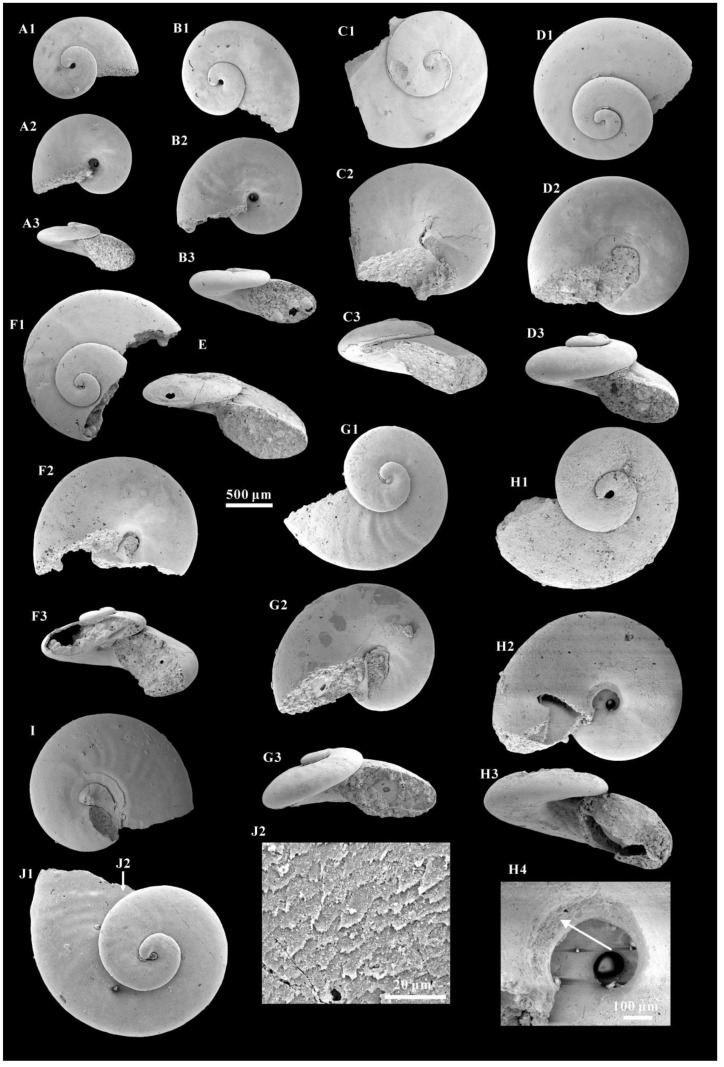
Different stages of *Aldanella attleborensis* from the Yanjiahe Formation in Yanjiahe Section. (**A**), internal molds, CUBar207-4; (**A1**) apical view, (**A2**) umbilical view, (**A3**) apertural view. (**B**), internal molds, CUBar208-2; (**B1**) apical view, (**B2**) umbilical view, (**B3**) apertural view. (**C**), internal molds, CUBar207-11; (**C1**) apical view, (**C2**) apertural view, (**C3**) umbilical view. (**D**), internal molds, CUBar206-12; (**D1**) apical view, (**D2**) umbilical view, (**D3**) apertural view. (**E**), internal mold, CUBar206-3, apertural view, showing the hollow. (**F**), internal molds, CUBar207-1; (**F1**) apical view, (**F2**) umbilical view, (**F3**) apertural view, showing the hollow internal mold. (**G**), internal molds, CUBar186-9; (**G1**) apical view, (**G2**) umbilical view, showing the well-preserved inner lip of aperture, (**G3**) apical view. (**H**), internal molds, CUBar206-9; (**H1**) apical view, (**H2**) umbilical view, (**H3**) apertural view, (**H4**) magnification of (**H2**), showing the spiral muscle scar. (**I**), internal molds, apical view, CUBar115-4. (**J**), internal molds, apical view, CUBar158-6; (**J2**) magnification of (**J1**), showing the laminar microstructure.

**Figure 3:**
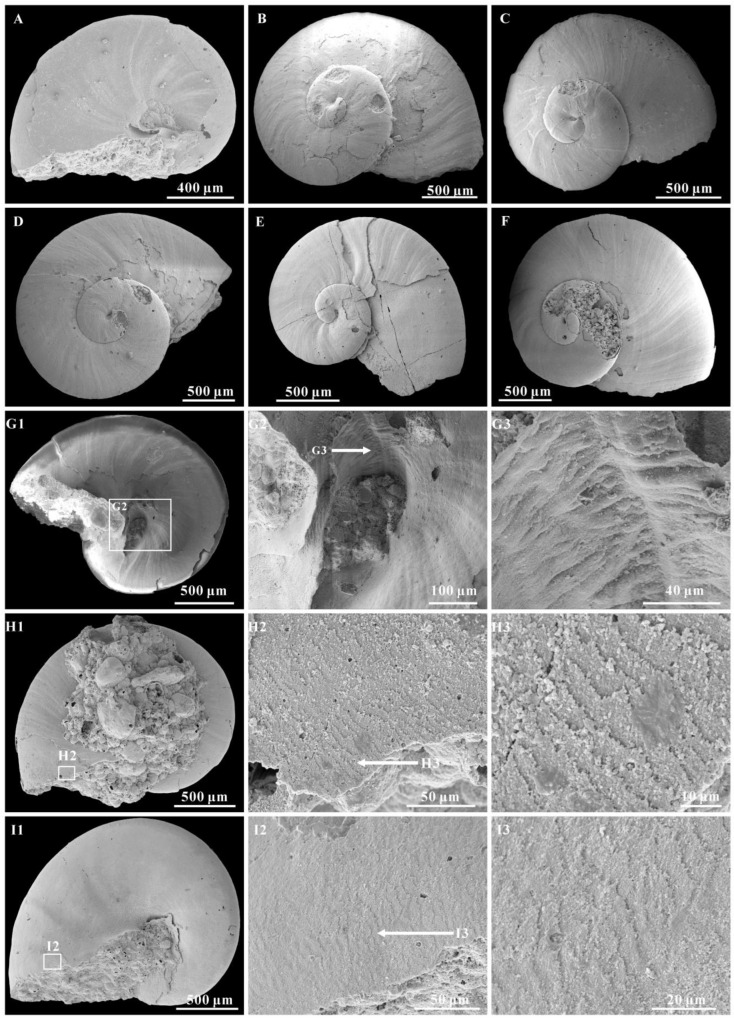
The growth lines and microstructure on the *Aldanella attleborensis*. (**A**), internal mold with external coating, umbilical view, CUBar11-10. (**B**), internal mold with external coating, apical view, CUBar249-7. (**C**), internal mold with external coating, apical view, CUBar249-8. (**D**), internal mold with external coating, apical view, CUBar249-17. (**E**), internal mold with external coating, apical view, CUBar250-3. (**F**), internal mold with external coating, apical view, CUBar250-4. (**G**), internal mold with external coating, umbilical view, CUBar98-10; (**G2**,**G3**) magnifications of (**G1**) showing the special ornamentation of umbilical areas. (**H**), internal mold, umbilical view, CUBar208-8; (**H2**,**H3**) magnifications of (**H1**) showing the laminar microstructure. (**I**) internal mold, umbilical view, CUBar208-4; (**I2**,**I3**) magnifications of (**I1**) showing the laminar microstructure. (**A**,**G**–**I**) from Yanjiahe Section, (**B**–**F**) from Gunziao Section.

**Figure 4:**
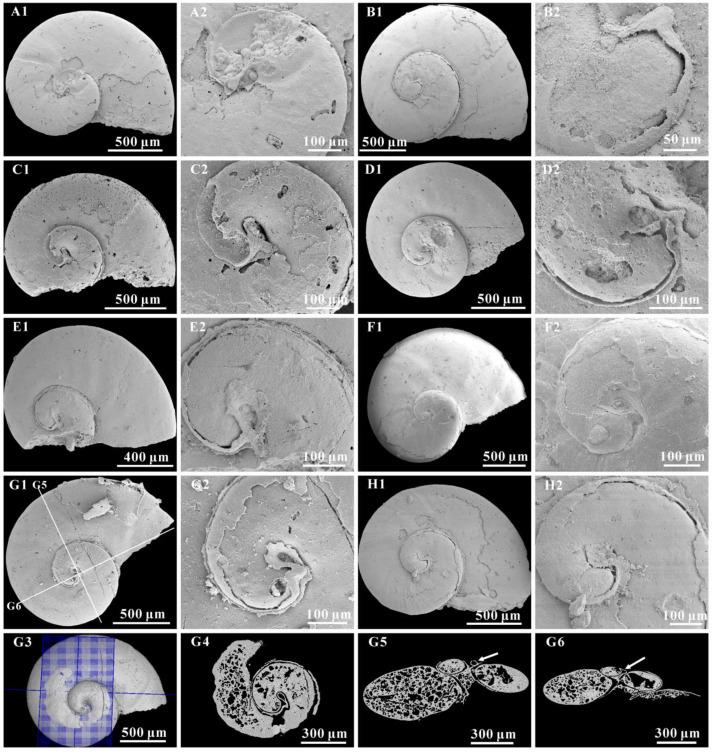
The mucro on the protoconch of *Aldanella attleborensis*. (**A**–**H**), internal molds with partial external coating, apical views, CUBar207-9, CUBar207-14, CUBar98-33, CUBar115-14, CUBar208-9, CUBar98-26, CUBar208-7, and CUBar22-4, respectively; (**A2**,**B2**,**C2**,**D2**,**E2**,**F2**,**G2**,**H2**) magnifications of (**A1**,**B1**,**C1**,**D1**,**E1**,**F1**,**G1**,**H1**), showing variation of mucros. (**G3**–**G6**), Micro-CT tomography of the (**G1**); (**G3**), 3D volume rendering; (**G4**), virtual intersection, indicated by the blue flat in (**G3**); (**G5**), virtual intersection traversed the mucro, indicated by the line in (**G1**); (**G6**), virtual intersection along the mucro, indicated by the line in (**G1**), white arrows indicate the mucro position. Specimens from the Yanjiahe Section.

**Figure 5:**
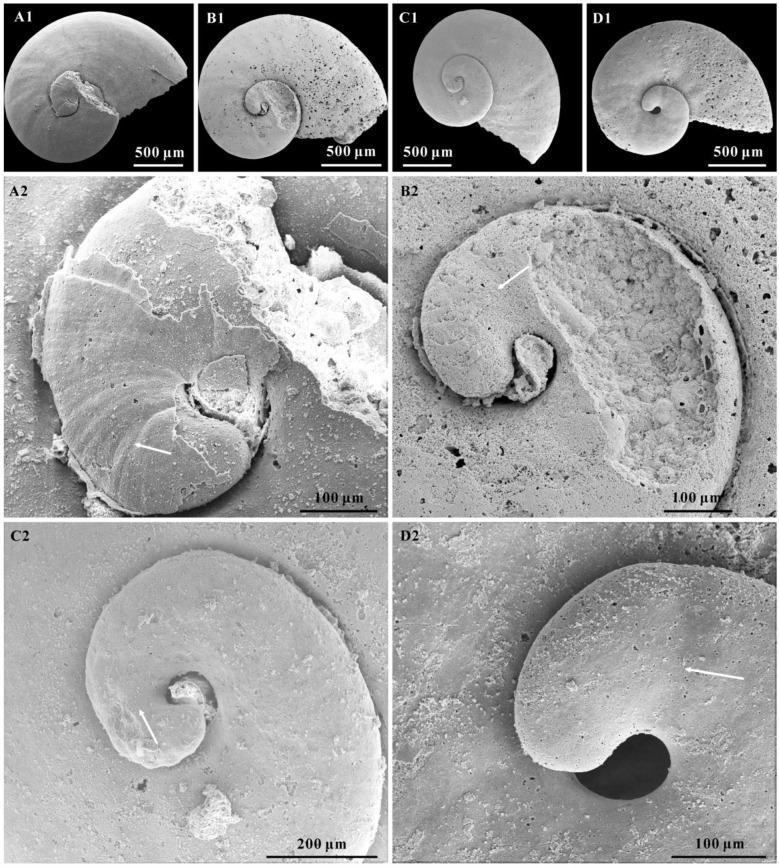
The protoconch of *Aldanella attleborensis*. (**A**), internal molds with external coating, apical views, CUBar79-26; (**A2**) magnification of (**A1**) showing the absence of growth lines on the protoconch. (**B**), internal molds, apical views, CUBar206-5; (**B2**) magnifications of (**B1**) showing the slight groove between the protoconch and teleoconch. (**C**), internal molds, apical views, CUBar14-12; (**C2**) magnifications of (**C1**) showing the slight groove between the protoconch and teleoconch. (**D**), internal molds, apical views, CUBar115-3; (**D2**) magnifications of (**D1**) showing the slight groove between the protoconch and teleoconch. White arrows indicate the position that distinguishes protoconch and teleoconch. Specimens from Yanjiahe Section.

*Aldanella attleborensis* Shaler and Foerste, 1888 [10]

(Figure 1, Figure 2, Figure 3, Figure 4 and Figure 5)

**Figure 6:**
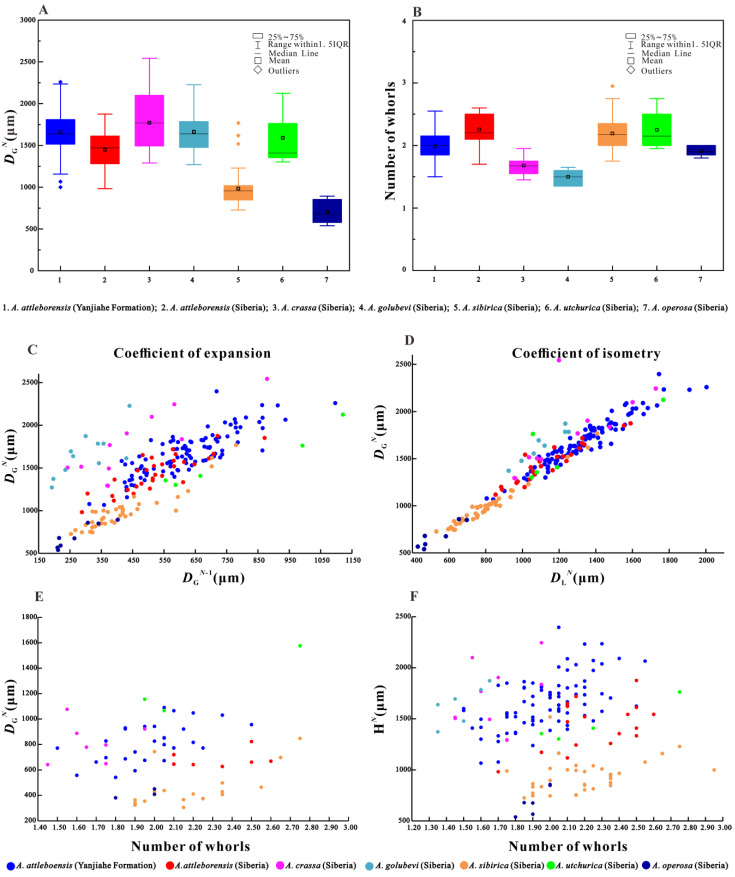
Morphological parameter analyses of *Aldanella*. (**A**), box-plot of greater shell diameter, including *A. attleborensis* (Yanjiahe Formation, *N* = 108), *A. attleborensis* (Siberia, *N* = 31), *A. crassa* (Siberia, *N* = 11), *A. golubevi* (Siberia, *N* = 11), *A. sibirica* (Siberia, *N* = 41), *A. utchurica* (Siberia, *N* = 5), *A. operosa* (Siberia, *N* = 8). (**B**), box-plot of number of whorls, including *A. attleborensis* (Yanjiahe Formation, *N* = 81), *A. attleborensis* (Siberia, *N* = 17), *A. crassa* (Siberia, *N* = 10), *A. golubevi* (Siberia, *N* = 7), *A. sibirica* (Siberia, *N* = 34), *A. utchurica* (Siberia, *N* = 4), *A. operosa* (Siberia, *N* = 6). (**C**), greater shell diameter/lesser shell diameter ratio showing the coefficient of isometry. (**D**), greater shell diameter at stage of *N* whorls/greater shell diameter at stage of *N*-1 whorls ratio, showing the coefficient of expansion. (**E**), linear regression between the number of whorls and the greater shell diameter. (**F**), linear regression between the number of whorls and the shell height. Siberian data from Parkhaev and Karlova [13], tables 1–6.

1888 *Pleurotomaria* (*Raphistoma*) *attleborensis* Shaler and Foerste, pp. 30–31, pl. 2, figure 2 [10].
1962 *Aldanella attleborensis* Vostokova, p. 66–67, pl. 2, figure 12a,b [23].
2011 *Aldanella attleborensis*; Parkhaev and Karlova, p. 1187, 1189, 1191, pl. 1, figures 1–6; pl. 2, figures 1–8; pl. 3, figures 1–10; pl. 4, figures 1–10; pl. 5, figures 1–8; pl. 17, figures 1 and 2 (and the synonymy list therein) [13].
2013 *Aldanella attleborensis*; Dzik and Mazurek, figures 2A–G, 3A–F and 5A–I [66].
2014 *Aldanella yanjiaheensis*; Guo et al., figure 4a [19].
2014a *Aldanella attleborensis*; Parkhaev, figure 1 [67].
2017 *Aldanella attleborensis*; Kouchinsky et al., pp. 347–351, figures 20A–F, 21A–G and 22A,B [21].
2020 *Aldanella attleborensis*; Steiner et al., figure 6A–E [46].
2021 *Aldanella attleborensis*; Guo et al., figure 3A–D [20].

Holotype. MCZ, no. 101331; United States, Nahant section; Lower Cambrian, Weymouth Formation. Designated by Shaler and Foerste [10].

Material. All the specimens (more than 100) studied herein were recovered from the base of Member 5 of the Yanjiahe Formation in the Yanjiahe and Gunziao sections. The original shells of *A. attleborensis* were dissolved by either diagenesis or acid maceration in the lab. Most specimens were preserved as phosphatic steinkerns exhibiting a hollow apical part and a stuffed apertural part (Figure 2E,F3), but some specimens had partial phosphatic external coatings. Because phosphate usually precipitates first from the internal surface of the shell/skeleton during diagenesis [68], internal molds are generally compactly preserved with a fine surface and occasionally with imprints of shell microstructures.

Diagnosis. Shell turbospiral, low-conical, and dextral. Umbilicus broad, upper whorl surface flattened, and periphery rounded. Aperture oblique oval. Expansion ratio of the shell moderate. Shell ornamented with growth lines. Protoconch small, cap-like with a mucro. Distinction between protoconch and teleoconch inconspicuous.

Description. Turbospirally coiled, dextral shell (Figure 2A1,B1,C1,D1,F1,G1,H1,I,J1), intermediate in size (approximately 2000 μm in diameter), consists of up to 2.6 tightly coiling whorls. The K^exp^ is moderate, (approximately 2.75), and the K^iso^ is small (approximately 1.27) (see Supplementary Table S1). The spire protrudes slightly above the surface of the last whorl (Figure 2A3,B3,C3,D3,E,F3,G3,H3). The upper whorl surface is flattened or slightly convex (Figure 2A3,D3,E,C3,G3). The periphery is rounded (Figure 2A2,B2,D2), and the base is slightly convex (Figure 2C3,D3,G3). The aperture is oblique oval in outline, with a width/height ratio of approximately 1.5. The umbilicus is wide (Figure 2A2,B2,C2,D2,F2,G2,H2), and some specimens exhibit opening umbilical perforations (Figure 2A,B,H).

The internal molds are mostly smooth (Figure 2A–D,F), or with more or less ornamentation of the transverse folds on the upper surface of last whorl (Figure 2G1,I,J). The transverse folds are clear on the middle of the whorl and fade away to the suture and periphery (Figure 2G1,I). However, some growth lines are clearly present on the external coating of the upper surface of the whorls and umbilical areas (Figure 3A–G). Growth lines occur from the protoconch margin and become clear at the aperture. They are expressed in an arched course and gradually become “S” shaped on the last whorl surface (Figure 3B–F). The spire of the internal mold is smooth.

The protoconch is cap-like, and faintly separates from the teleoconch. It is approximately 100–200 μm in length and always bears a mucro (Figure 3D–F and Figure 4A–H). In some specimens, the protoconch could be demarcated from the teleoconch by a slight groove (Figure 5B–D) or the absence of growth lines (Figure 5A).

A hollow space exists between the internal mold and external coating (Figure 3A,D–G and Figure 4G3–G6), and it may reflect the morphological space of the original shell. The calcareous shells were dissolved away after acid maceration in the lab or during diagenesis. The shell wall is thin (approximately 10 μm) compared with the diameter of the whorl and always thickens in the umbilical area (Figure 4G5).

Remarks. *Aldanella attleborensis* was originally described as *Pleurotomaria* (*Raphistoma*) *attleborensis* by Shaler and Foerste [10] from the Cambrian strata of Massachusetts. When the genus *Aldanella* was established by Vostokova in 1962 [23] based on samples from the Siberian Platform, *Pleurotomaria* (*Raphistoma*) *attleborensis* was designated as its type species. Owing to the specific morphological features, *Aldanella* can be easily distinguished from other Cambrian mollusks. However, the endemic features and morphologic variations have led to an over-splitting taxonomy of *Aldanella*. After several rounds of revisions [13,39], *A. kunda*, *A. rozanovi*, *A. costata*, *A. patelliforma*, and *A. yanjiaheensis* have been regarded as junior synonyms of *A. attleborensis* based on the similar diagnostic features that were considered to be intraspecific variations. However, a detailed analysis of *A. yanjiaheensis* based on the materials from its type locality has not been performed.

In South China, specimens of *Aldanella* were first recovered from the Yanjiahe Formation of the Three Gorges area by Chen [33], and a new species, *Aldanella yanjiaheensis*, was established because these specimens were different from *Aldanella* ex. gr. *attleborensis* by having a smaller shell diameter. On account of the similarities in morphology, Landing [39] considered *A. yanjiaheensis* as a junior synonym of *A. attleborensis*. Although *Aldanella* has been occasionally reported in many locations in China [9,19,20,44,45,46,47,48,49,69], the scarcity of specimens has limited paleontologists to give a detailed taxonomic revision.

In this study, 109 specimens of *Aldanella* collected from the ‘*A. yanjiaheensis*’ type locality were studied and measured (Supplementary Table S1). All the specimens are dextral, and low-conical, the shells diameter ranges from 1000 to 2400 μm, and the expansion ratio is moderate. The protoconch of the specimens is small, about 100–200 μm long, and always bears a mucro. The shell whorl surface is more flattened, and ornamented with traverse folds or growth lines. Except for the larger shell diameter, the morphological features and ornamentation of *Aldanella* specimens from the Yanjiahe Formation are consistent with *A. attleborensis*. So, our current study formally confirmed that the species *A. yanjiaheensis* Chen, 1984 [33] is a junior synonym of *A. attleborensis* [13,39].

*Aldanella attleborensis* differs from *A. golubevi* by the dextral shell and smaller expansion ratio (Figure 6C); from *A. sibirica* by the larger (6A) and higher (6F) shell, and the more flattened upper whorl surface; from *A. crassa* by the smaller shell diameter (6A), smaller expansion ratio (6C), and higher whorl number of the shell (6B); from *A. operosa* by the absence of septum structure on the apical part, and much larger shell diameter (6A); and from *A. utchurica* by its much lower shell.

## 4. Discussion

### 4.1. Shell Microstructures of Aldanella attleborensis

Shell microstructures could be remarkably well preserved on the Cambrian mollusk shells [20,70,71], and numerous types of shell microstructures have been identified, such as prismatic, nacre, crossed lamellar, and foliated. However, only convex polygons [21,40,72,73,74,75] and lamellar microstructures [70] have been occasionally reported on internal molds of *Aldanella*.

The convex polygons appear on the spire of internal mold surface, and are about 20–60 μm in diameter. The convex polygons are varying in shape, mainly circular or nearly circular, and always separated by shallow grooves [21,72,73,75]. Conversely, in some literature, the space between convex polygons is large and appears as concave polygons. Hence the convex and concave polygons seem to alternate on the spire of internal mold of *Aldanella* [40,74]. Similar convex polygonal impressions were described on the apex of *Oelandiella* and *Securiconus*, and were mostly interpreted as imprints of the prismatic layer [40,73,75]. The imprints of the prismatic layer always preserved as negative or positive reliefs on the molluscan internal molds [71]. Different to the prismatic layer imprints, the convex polygons of *Aldanella* are restricted to the apical areas [21,40,72,73,74,75] and no tubercles in the polygons. In addition, the polygons that are interpreted as imprints of the prismatic shell layer are much smaller (10–20 μm), although the varied size of convex polygons has been attributed to the growth stages and competition for space [73]. The convex polygons of *Aldanella* have been interpreted as the impressions of cells of the outer epithelium [13,74], but this view remains to be investigated.

Inner layer laminar microstructure, the only preserved microstructure on the *A. attleborensis* of the Yanjiahe Formation, appears on the internal mold surface near the aperture (Figure 2J2 and Figure 3H,I). Different to the laminar microstructures of *Pojetaia* with a rounded edge, the lamella of herein shows two different forms: straight edge (Figure 3H) or sharp-toothed edge (Figure 2J2 and Figure 3I). The orientation of the lamella points to the suture on the upper whorl surface and to the umbilicus on the base. This type of microstructure is common in Cambrian mollusks, such as *Anabarella*, *Watsonella*, *Fordilla*, *Pojetaia*, and *Pelagiella* [20,70,71,76], and is interpreted as a foliated aragonite [70].

### 4.2. Muscle Scars

Muscle scars provide important information to study the morphology and phylogeny of ancient univalved mollusks. Compared to most cap-shaped mollusks that have paired muscle scars on the dorsal margin, apical area, or sub-apical area [77,78], the muscle scars are always difficult to see in coiled gastropod shells where the muscle attachment site is always restricted on the umbilical area, as a columellar scar [79].

On the internal mold surface of *A. attleborensis*, a spiral structure is found on the umbilical area (Figure 2H4). The spiral structure appears as about three-quarters of a whorl and vanishes near the aperture. The spiral structure is ribbon-shaped, and the width increases in the progress of growth. A similar structure has been interpreted as columellar muscle attachment on the shell umbilicus of *A. rozanovi* and *A. kunda* (junior synonyms of *A. attleborensis*) [26,40]. The difference is that on *A. rozanovi*, the spiral structure consists of honeycomb patterns with a diameter up to 6–8 μm. Isakar and Peel [40] urged caution to compare the honeycomb patterns on *A. rozanovi* umbilicus [26] with gastropod retractor muscles, because the honeycomb pattern is similar to the convex polygons on the spire of the internal molds. However, they ignored the fact that the convex polygons are much larger than the honeycomb patterns. In addition, on the apical surface of *A. kunda*, there exists an equivocal spiral ridge interpreted as the trace of a spirally migrating shell attachment muscle [40]. In Paleozoic helically coiled gastropods, ribbon-like muscle scar appeared on the umbilical area of internal molds, such as Ordovician *Tropidodiscus* [80], Silurian *Megalomphala* [81], Carboniferous *Porcellia* [82], and *Bellerophon* [81], but it is rare in Cambrian gastropods. The muscle scar on *Tropidodiscus* appeared on the umbilical shoulder and was preserved as a slightly depressed zone [80]. The position and the shape of *Tropidodiscus* muscle scar is consistent with that of *Aldanella*. The muscle scar of *Megalomphala* was preserved as smooth area on the umbilical wall of the internal mold, separated by two spiral ridges from the internal mold [81]. On the *Porcellia*, a single ribbon-like muscle scar is longer and narrower, and lies on the basal surface of the whorl [82]. The muscle scar of *Bellerophon* is located on the umbilical shoulder, but the difference is that *Bellerophon* has a pair of muscle scars and the scars were preserved as a raised zone [81].

Besides *Aldanella*, similar ribbon-like structures also have been described on Cambrian coiled mollusk *Pelagiella* [63,83,84]. That is an important characteristic to support these genera as gastropods [26,27]. Comparing the ribbon-like structure position and shape of *A. attleborensis* with that of the muscle scars on the upper Paleozoic gastropods, as well as the columellar muscles on living spirally coiled gastropods, it is herein considered that the spiral structure on the umbilicus of *Aldanella* is the shell muscle attachment area, although some of upper Paleozoic gastropods have a pair of muscle scars.

### 4.3. Systematic Position of Aldanella

The systematic position of the genus *Aldanella* is controversial, and has been suggested to gastropods [13,24,25,26,27], helcionelloids [40,85], hyoliths [66], or polychaetes [86,87]. The phosphatic preservation of the shell and the absence of the soft body provide extremely limited evidence to study the phylogeny and affinity of this genus. The imprints of the shell microstructures and the shell muscle attachments have been recognized as significant information to reveal the systematic position of early mollusks [20,70,71,88,89], and therefore, it is necessary to research these features on *Aldanella*.

The presence of laminar microstructure on the internal molds of *A. attleborensis* provides crucial information to analyze its systematic position. As the laminar microstructure reported in *Pelagiella* has been suggested to be nacreous by Thomas et al. [27], the presumption that Pelagielliformes microstructure is comparable to that of hyoliths in lacking nacre is inappropriate. Most importantly, the operculum, preserved in hyoliths, has never been found in Pelagielliformes. Apparently, *Aldanella* is not a hyolith. The septa presented in the initial shell of *A. operosa* exclude the possibility that *Aldanella* is a polychaete [26]. Furthermore, laminar microstructure was never discovered in polychaetes [90]. In addition, *Aldanella* was suggested as helcionelloids because they share an endogastric shell. However, the muscle scar preserved on the umbilicus area of *A. attleborensis* is more similar to that of Paleozoic gastropods than the paired muscle scars of helcionelloids. Hence, according to the muscle scars similarity of *Aldanella* and upper Paleozoic gastropods in position and shape, the appearance of laminar microstructure on the *Aldanella* shell, the presence of protoconch and regular septa in the initial part of the shell, as well as the asymmetrically turbospiral shell, *Aldanella* is more likely to be a gastropod than helcionelloids, hyoliths, or polychaetes.

### 4.4. Ontogeny of Aldanella attleborensis

Ontogenetic analysis is a useful method for studying the origin and early evolution of gastropods. Based on the fossil materials of Paleozoic, many achievements on gastropod ontogeny have been made [91,92]. However, the ontogeny of early Cambrian gastropods, such as *Aldanella* and *Pelagiella*, is rarely systematically studied due to limited specimens. The shell of *Aldanella* was ontogenetically identified in two (protoconch and teleoconch) or three (protoconch I, protoconch II, and teleoconch) developmental stages [93].

The morphology of *A. attleborensis* analyzed in this study can be compared with Siberian materials, and the protoconch and teleoconch of the shell is faintly separated. The protoconch of *A. attleborensis* is small, approximately 100–200 μm in length. The small size of the protoconch may indicate the existence of the planktonic larval stage during development [24,91,93,94], which is consistent with its worldwide distribution in the lower Cambrian strata. The protoconch always bears a mucro on those specimens preserved with external coating, but the protoconch of the internal mold is smoothly rounded (Figure 4A2,H2). This is a unique character that has been found on the external coating of *Aldanella* [66,72]. The size and shape of the mucro is variable, and it looks like a short column (Figure 4C2,E2,F2) or blunt cusp (Figure 4A2,B2,D2,G2). As shown in the Micro-CT images (Figure 4G4–G6), when the original shell is dissolved, the mucro is expressed as a hollow space. This means that the mucro is an original solid protrusion on the protoconch. Moreover, the mucro has no surface ornamentation. Although the function of the mucro remains unknown, there is no ground to conclude that it may represent a discrete stage of larval shell development, as suggested by Dzik [95] and Thomas et al. [27].

Most of the specimens studied herein were partially broken, especially at the protoconch and aperture. Nevertheless, the well-preserved inner lip of the aperture on some specimens with phosphatic coating (Figure 2G2 and Figure 3A) indicates that the steinkerns almost represent complete specimens, not just broken off pieces of larger shells [66]. The specimens of *A. attleborensis* described herein range from 1067 μm to 2397 μm in diameter (Figure 6A), and from 1.50 to 2.55 in the number of whorls (Figure 6B). The wide range of sizes and whorl numbers of the shells suggests that both juvenile and adult stages are preserved. In addition, the box-plot of shell diameter and whorl numbers showed that the adult shell size mainly ranges from 1400 μm to 1900 μm, and the whorl numbers correspond to approximately 2. The strong linear regressions of the expansion and isometry rates (Figure 6C,D) indicate that the shell tube width (the straight-line distance from suture to periphery) of *A. attleborensis* constantly increases. Owing to the turbospirally coiled shell, the length of shell tube (the helical-line distance from protoconch to aperture) is difficult to measure. Compared with the diameter of the shell, the number of whorls could be more accurate in estimating the longitudinal growth rate of the shell tube. Although the general trend is positive, the weak linear regression of the shell diameter and number of whorls (Figure 6E) suggests that the final shell size is not strongly correlated with the whorl number, and the length of shell tube of the whorl is allometric growth relative to its width. In addition, the shell height had a weak relationship with the number of whorls (Figure 6F). The shell height might be more affected by the obliquity and oblateness of shell tube cross section.

### 4.5. Stratigraphic Implications of Aldanella attleborensis

With the progress of Cambrian stratigraphy subdivision, more than half of the Global Boundary Stratotype Section and Points (GSSPs) of Cambrian stages have been ratified. However, due to high levels of endemism and the facies-restricted distribution of fossils, as well as the absence of the pelagic skeletonized metazoans used for global correlation, formal ratification of Stage 2, Stage 3, and Stage 4 is difficult [41,96]. Nevertheless, SSFs play an important biostratigraphic role for the subdivision and correlation of pre-trilobitic (Terreneuvian) Cambrian strata [4,9,15,16,17,18]. For example, the widely distributed *A. attleborensis* or *W. crosbyi* both have been suggested as a potential marker for defining the base of Cambrian Stage 2.

In this study, numerous specimens of *A. attleborensis*, co-occurring with *W. crosbyi*, have been discovered from Member 5 of the Yanjiahe Formation of the Three Gorges area, South China. Besides the Yanjiahe Formation, *A. attleborensis* was also discovered from the uppermost Huangshandong Member (Tianzhushan Member) of the Dengying Formation in Tianzhushan of the Three Gorges area [47]. The thickness of the Huangshandong Member is extremely varied at different sections in the Three Gorges area (0.1-8 m) [43,44,97], and the SSF assemblages are often mixed [43,44], or SSF zone 1 and 2 could be recognized [46,97]. However, besides *A. attleborensis*, *W. crosbyi* was also found in the uppermost Tianzhushan Member from the Tianzhushan Section [97], Huangshandong Section [43,97], and Songlinpo Section (unpublished data). This may indicate that the SSF zone 3 occasionally exists in the uppermost Tianzhushan Member at some sections with a relatively thick unmixed sequence of this lithological unit.

The similar phenomenon also appears in southern Shaanxi. The Kuanchuanpu Formation is well-exposed in southern Shaanxi and contains abundant SSFs. Nevertheless, scarce materials of *A. attleborensis* and *W. crosbyi* were only recovered in the uppermost Kuanchuanpu Formation at the Yuanjiaping Section of Ningqiang County [43]. This indicates that the sediments containing SSF zone 3 are rare in the Kuanchuanpu Formation of southern Shaanxi. In addition, *Aldanella attleborensis* was discovered from the Dahai Member of the Zhujiaqing Formation in Eastern Yunnan [9,42], but its first occurrence horizon is much higher than that of *W. crosbyi* [42].

Using *A. attleborensis* as a biostratigraphic marker, Member 5 of the Yanjiahe Formation, uppermost Tianzhushan Member, uppermost Kuanchuanpu Formation, and the Dahai Member can be well correlated. Therefore, the FAD of *A. attleborensis* has a potential to correlate the pre-trilobitic strata in South China. In addition, *A. attleborensis* was documented in the upper part of the Yurtus Formation, Aksu-Wushi region, Tarim [48,49,69], but the age of the uppermost Yurtus Formation has been suggested to be Qiongzhusian equivocally [49]. Therefore, in Tarim the detailed stratigraphic correlation still needs to be further confirmed.

**Figure 7:**
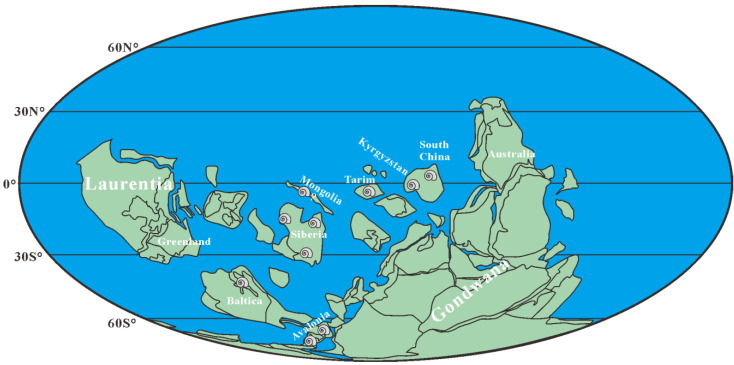
Paleogeographical distribution of *Aldanella attleborensis* from Cambrian Stage 2. Paleogeographic map modified from [98] and [20]; the distribution data from [9,13,21,33,39,40,49,99,100] and herein.

With the exception of South China, *Aldanella attleborensis* is also widely distributed in the pre-trilobitic strata of most paleocontinents, such as Avalonia, Baltica, Siberia, etc. (Figure 7). The materials of *A. attleborensis* in Siberia are extremely rich, and its stratigraphic range were suggested to be the whole Tommotian [13]. However, *A. attleborensis* may appear much lower than *W. crosbyi* in Siberia [21,50] and the hiatus between the Pestrotsvet Formation and the underlying Ust-Yudoma Formation [101] makes it difficult to evaluate the accuracy of the FAD [12]. The FADs of *W. crosbyi* and *A. attleborensis* in Avalonia correspond to the base of the Tommotian, but the temporal range of *W. crosbyi* and *A. attleborensis* is much longer and their LADs (last appearance data) might not be limited to the Tommotian [41]. In western Mongolia, the original description of *W. crosbyi* assemblage zone suggested a Tommotian age [100], but it was revised to be pre-Tommotian in age by Smith et al. [102] based on chemostratigraphic data. *Aldanella* in western Mongolia was uncommon, appeared much higher than *W. crosbyi*, and only one specimen, assigned to *Aldanella* sp., was reported from the probable Botoman Stage [100]. Although this indeterminate species has been revised to *A. attleborensis* [13], the horizon and taxonomy of *A. attleborensis* in western Mongolia [100] is questionable [21]. *Aldanella attleborensis* has been reported in the Kestla Member of the Lontova Formation in northern Estonia [40], and its age is limited to the Tommotian [67,99].

**Figure 8:**
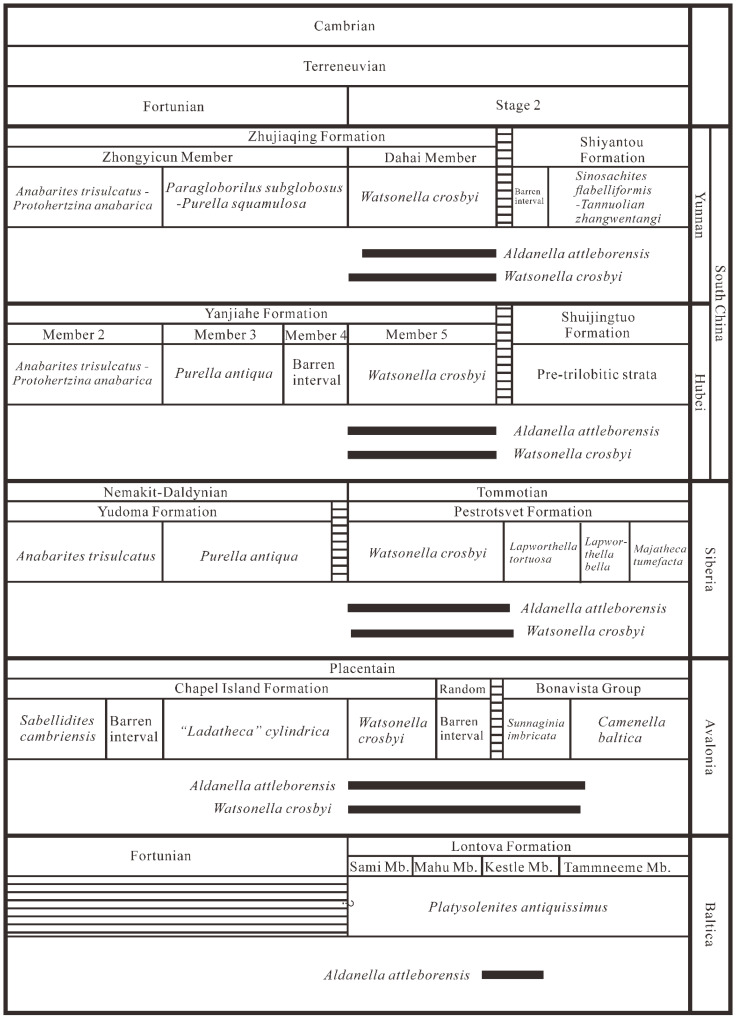
Global chronostratigraphic correlation chart of Fortunian and Stage 2, Terreneuvian. Biostratigraphic and biozone data from [9,12,20,21,40,41,67,104].

In summary, *A. attleborensis* is one of the most widely distributed SSFs and is an important fossil for both regional and global correlation of Cambrian Terreneuvian (Figure 8). Based on its wide geographic distribution and distinct turbospirally coiled shell, *A. attleborensis* has the potential to be an index fossil for defining the base of Cambrian Stage 2 [13,14,67]. In addition to *A. attleborensis*, another mollusk, *W. crosbyi*, which is broadly similar in stratigraphic distribution to *A. attleborensis*, such as South China, Siberia, Avalonia, Australia, Armorican France, and Mongolia (see [12,20] and references cited therein), has also been suggested as a potential marker for defining the base of Cambrian Stage 2 [12,103].

## 5. Conclusions

Numerous specimens of *Aldanella* were collected from Member 5 of the Yanjiahe Formation at the Yanjiahe and Gunziao sections in the Three Gorges area, South China. This study shows that *A. yanjiaheensis* is morphologically consistent with *A. attleborensis* in many aspects, such as the small turbospiral shell, moderate expansion rate, up to 2.5 tightly coiled whorls, oblique oval aperture, and growth lines occurring on the shell. Our work formally confirms that the species *A. yanjiaheensis* Chen, 1984 is a junior synonym of *A. attleborensis*.

Two developmental stages, protoconch and teleoconch, of the shell of *A. attleborensis* were identified. The small protoconch of the shell bears a distinct mucro. The strong linear regressions of the expansion and isometry rates indicated that the shell tube width constantly increases. However, the weak linear regression of the shell diameter and number of whorls suggests that the shell tube of the whorl is allometric growth. The spiral muscle scar structure further confirms that *Aldanella* is more likely to be a gastropod.

*Aldanella attleborensis* widely occurred in Cambrian paleocontinents (South China, Mongolia, Siberian, Baltica, Avalonia, etc.) from low to high paleolatitudes. The stratigraphic range of *A. attleborensis* indicates that it was mainly restricted to Cambrian Stage 2. *Aldanella attleborensis* can be easily distinguished by its characteristic features from other congeneric species. Therefore, similar to *W. crosbyi*, the FAD of *A. attleborensis* could also be a potential candidate marker for defining the base of Cambrian Stage 2. The co-occurrence of *A. attleborensis* and *W. crosbyi* at the base of Member 5 of the Yanjiahe Formation indicates that Member 5 belongs to Cambrian Stage 2.

## Data Availability

Not applicable.

## Supplementary Materials

The following supporting information can be downloaded at: https://www.mdpi.com/article/10.3390/biology12020261/s1, Table S1: The datum of *Aldanella attleborensis* measurements.
